# Don't stop your heart in front of your family: family as a bystander is associated with poor outcome of bystander-witnessed, bystander-CPR-performed out-of-hospital cardiac arrest

**DOI:** 10.1186/cc10879

**Published:** 2012-03-20

**Authors:** H Inaba, K Takase, T Nishi, T Kamikura, Y Wato, H Hamada

**Affiliations:** 1 Kanazawa University Graduate School of Medicine, Kanazawa, Japan; 2 Kanazawa Medical University, Uchinada, Japan; 3 Suzu General Hospital, Suzu, Japan

## Introduction

Early CPR with a considerable quality is essential for survival from out-of-hospital cardiac arrest (OHCA). This study was conducted to test our hypothesis that the relation of the bystander to the victim may affect the outcomes of OHCAs.

## Methods

From a Japanese nationwide database for 431,968 OHCAs that occurred from January 2005 to December 2008, we extracted and then analyzed 45,248 bystander-witnessed, bystander-CPR-performed OHCAs without any involvement of physicians. Backgrounds, characteristics and outcomes were compared among the three groups of OHCAs categorized by the bystander's relation to victims. Multiple logistic regression analysis was applied to clarify if the relation may affect the 1-month survival with favourable neurological outcomes.

## Results

When the bystander was family, CPR was more frequently initiated following telephone-assisted instruction and the interval between collapse and bystander CPR was significantly prolonged. Univariate analysis followed by multiple logistic regression analysis revealed that family as a CPR performer significantly decreases the 1-month survival with favourable neurological outcomes. See Tables 1 and 2.

**Table 1:** Backgrounds, characteristics and outcomes of OHCAs with reference to relation of bystander to victim

	Relation of bystander to victim			
		
Background, characteristics and outcome	Family (*n *= 25,119)	Friends, colleagues and passers-by (*n *= 5,191)	Others (*n *= 14,938)	*P *value
Patient's age, median (25 to 75%)	77 (66 to 84)	61 (50 to 73)	84 (75 to 90)	<0.001
Sex - male (%)	61.6	76.8	44.7	<0.001
CPR following telephone CPR (%)	75.1	42.7	36.1	<0.001
Initial rhythm shockable (%)	16.3	33.4	9.8	<0.001
Tune intervals, minutes, median (25 to 75%)				
Collapse-call	2 (0 to 5)	2 (0 to 4)	2 (0 to 5)	<0.001
Collapse-bystander CPR	3 (1 to 6)	1 (0 to 4)	0 (0 to 2)	<0.001
Call arrival at patient	8 (6 to 11)	8 (6 to 11)	8 (6 to 10)	<0.001
Outcomes				
1-month survival (%)	8.1	17.2	9.2	<0.001
1-month survival with favorable	4.0	11.9	4.8	<0.001
neurological outcomes (%)				

**Table 2:** Relation of bystander to victim as a factor associated with 1-month survival of bystander-witnessed OHCAs having bystander CPR

	Adjusted odds ratio (95% confidence interval)		
	
Factor	Bystander-witnessed OHCAs with bystander CPR	Of presumed cardiac etiology	Of presumed cardiac etiology with shockable initial rhythm
Etiology of arrest			
Presumed cardiac	1.39 (1.24 to 1.55)	Undefined	Undefined
Noncardiac	Reference		
Initial rhythm			
Shockable	4.38 (3.95 to 4.85)	4.82 (4.29 to 5.42)	Undefined
Nonshockable	Reference	Reference	Reference
Patient's age	0.97 (0.97 to 0.98)	0.97 (0.97 to 0.97)	0.98 (0.97 to 0.98)
Sex			
Male	1.14 (1.02 to 1.26)	1.16 (1.02 to 1.32)	1.07 (0.90 to 1.26)
Female	Reference	Reference	Reference
Relation of bystander to victim			
Family	Reference	Reference	Reference
Friend, colleague and passers-by	1.70 (1.49 to 1.95)	1.40 (1.19 to 1.64)	1.61 (1.42 to 1.81)
Others	1.59 (1.42 to 1.78)	1.46 (1.27 to 1.68)	1.32 (1.10 to 1.59)

## Conclusion

Despite educational efforts, most family members do not appear to be good CPR performers. The first responder system that enables a good CPR performer to reach the scene quickly may be needed for OHCAs witnessed by the family.

